# The Future of Breast Cancer Organized Screening Program Through Artificial Intelligence: A Scoping Review

**DOI:** 10.3390/healthcare13040378

**Published:** 2025-02-10

**Authors:** Emma Altobelli, Paolo Matteo Angeletti, Marco Ciancaglini, Reimondo Petrocelli

**Affiliations:** 1 Department of Life, Health and Environmental Sciences, Section of Epidemiology and Biostatistics Unit, University of L’Aquila, 67100 L’Aquila, Italy; paolomatteo.angeletti@gmail.com; 2 Cardiovascular Department, UO of Cardiac Anesthesia of the IRCCS Humanitas Research Hospital, 20089 Rozzano, Italy; 3 Department of Life, Health and Environmental Sciences, Section of Clinical and Molecular Medicine, University of L’Aquila, 67100 L’Aquila, Italy; marco.ciancaglini@univaq.it; 4 Public Health Unit, Azienda Sanitaria Regionale Molise, 86100 Campobasso, Italy; r.petrocelli@libero.it

**Keywords:** breast cancer, artificial intelligence, screening program, scoping review

## Abstract

**Objective**: The aim of this scoping review was to evaluate whether artificial intelligence integrated into breast cancer screening work strategies could help resolve some diagnostic issues that still remain. **Methods**: PubMed, Web of Science, and Scopus were consulted. The literature research was updated to 28 May 2024. The PRISMA method of selecting articles was used. The articles were classified according to the type of publication (meta-analysis, trial, prospective, and retrospective studies); moreover, retrospective studies were based on citizen recruitment (organized screening vs. spontaneous screening and a combination of both). **Results**: Meta-analyses showed that AI had an effective reduction in the radiologists’ reading time of radiological images, with a variation from 17 to 91%. Furthermore, they highlighted how the use of artificial intelligence software improved the diagnostic accuracy. Systematic review speculated that AI could reduce false negatives and positives and detect subtle abnormalities missed by human observers. DR with AI results from organized screening showed a higher recall rate, specificity, and PPV. Data from opportunistic screening found that AI could reduce interval cancer with a corresponding reduction in serious outcome. Nevertheless, the analysis of this review suggests that the study of breast density and interval cancer still requires numerous applications. **Conclusions**: Artificial intelligence appears to be a promising technology for health, with consequences that can have a major impact on healthcare systems. Where screening is opportunistic and involves only one human reader, the use of AI can increase diagnostic performance enough to equal that of double human reading.

## 1. Introduction

Breast cancer (BC) is a global health problem and is one of the principal causes of morbidity and mortality in females. In women, breast cancer (BC) represents the second highest cause of death, with 2 million new cases in 2020, and 80% of patients with BC are individuals aged >50 [1].

The risk of developing breast cancer increases 1.5% at age 40, 3% at age 50, and more than 4% at age 70 [2]. In 2030, the worldwide number of new cases diagnosed will reach 2.7 million annually, while the number of deaths will be 0.87 million [3]. The estimated global economic cost of cancers from 2020 to 2050 will be $25.2 trillion in international dollars (at constant 2017 prices), equivalent to an annual tax of 0.55% on global gross domestic product. The five cancers with the highest economic costs are tracheal, bronchus, and lung cancer (15.4%); colon and rectum cancer (10.9%); breast cancer (7.7%); liver cancer (6.5%); and leukemia (6.3%) [4,5].

The effectiveness of screening in reducing BC mortality is well-known. In addition, screening programs offer the advantage of early lesion detection, enabling their management before progression and worsening. Several efforts have been made to institutionalize population-based screening programs in many countries worldwide. However, in some countries, there is a lack of population-based (PB) screening programs [6,7].

Despite the advantages of current screening mammography, it is known that it is associated with a high risk of false positives and false negatives; therefore, the diagnostic accuracy must be improved. The introduction of artificial intelligence (AI) is becoming an important application in medical technologies [8,9,10]. Recently, a prevalent field of application concerns the combination of AI and radiological evaluation in mammographic screening.

The current research among researchers is to evaluate whether AI could help to reduce missed cancers and false positives as well as detect cancers at earlier stages [11].

The aim of this scoping review was to evaluate whether artificial intelligence integrated into breast cancer screening work strategies could help resolve some diagnostic issues that still remain.

## 2. Methods

**Figure 1:**
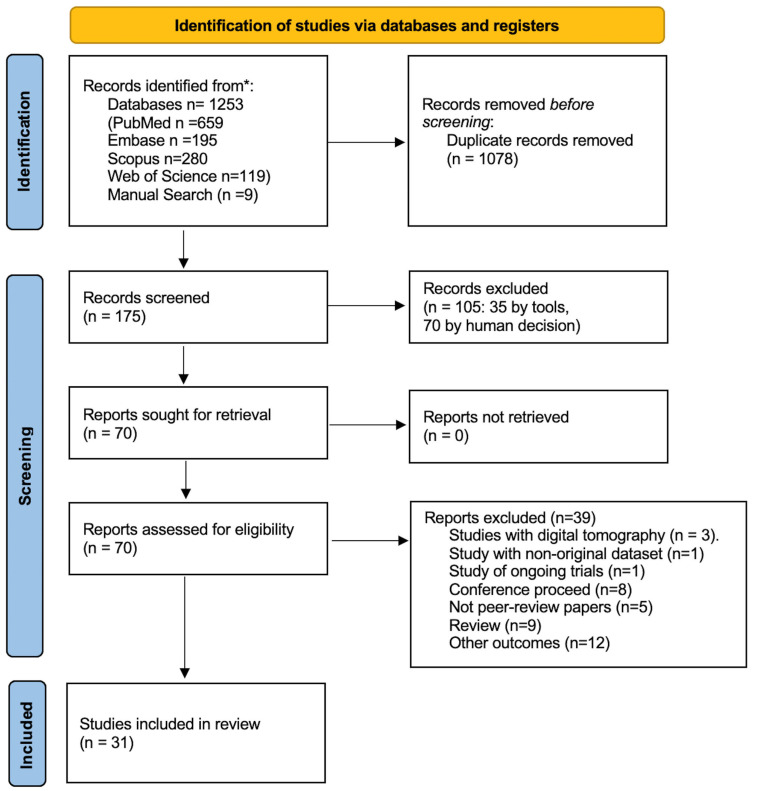
PRISMA flowchart.

The following databases were consulted: Embase, PubMed, Web of Science, and Scopus. The search keys are reported in the Supplementary Materials. The keywords used for each database are reported in Supplementary Table S1. The literature research was updated to 28 May 2024. Articles published in English in the last 10 years were included. Conference proceedings, articles in preprints, and in general publications not subjected to peer review were excluded. The PRISMA Figure 1 method of selecting articles was used [12]. The quality of the primary studies was tested using the following scales: AMSTAR 2 by Shea et al. [13] for meta-analyses and systematic reviews (Supplementary Table S2); the Cochrane Clinical Trial for randomized studies (Supplementary Table S3) [14]; and the Newcastle–Ottawa for observational studies (Supplementary Table S4) [15]. Finally, a checklist was applied, according to Tricco et al., for the final control of this scoping review (Supplementary Table S5) [16].

**Figure 2:**
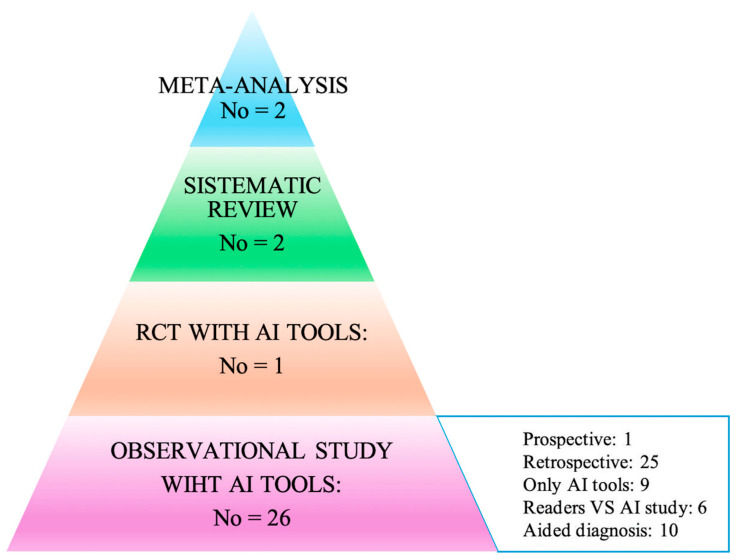
Hierarchy results of the literature research using the evidence-based pyramid with artificial intelligence (adapted from Bellini et al. and Murad et al.) [17,18].

The articles were classified in two ways: the first according to the type of publication (meta-analysis, trial, prospective, and retrospective studies), and the second based on the way in which AI was used within the individual works. In particular, attention was paid to the acquisition method of the primary data (study design), and consequently, how AI was used to analyze the data itself to arrive at the diagnoses: (i) AI only on datasets; (ii) AI toward readers; (iii) AI to support readers. All of this is summarized in the hierarchical model of the pyramid of scientific evidence as reported in Figure 2 [17,18].

Moreover, retrospectives studies were based on citizen recruitment (organized screening vs. spontaneous screening and a combination of both) (Table 1 and Table 2).

## 3. Results

The results of the literature research (Figure 1 and Figure 2) identified two meta-analyses, one systematic review, one trial, and eighteen cohort studies, of which one was prospective and seventeen retrospective, as highlighted in Figure 2.

### 3.1. Meta-Analyses

The literature review identified two meta-analyses of interest.

The first by Hickman et al. [19], conducted on 14 studies, highlighted how the use of artificial intelligence software improved the diagnostic accuracy (Table 1). AI demonstrated an effective reduction in the radiologists’ reading time of radiological images, with a variation from 17 to 91%. Furthermore, missed cancers by the readers were diagnosed by AI from 0% to 7%. These results are reported in Table 1.

The second by Yoon [20] shared the results of the previous meta-analysis and also included four digital breast tomography (DTB) studies. For both mammography and DBT, the performance of AI appeared to be greater than that of the human readers (Table 1).

### 3.2. Systematic Reviews

Sixteen studies were included in Schopf’s review [21]. The purpose of the review was to analyze the use of AI alone or AI in conjunction with clinical risk tools for breast cancer. Although there were no cumulative data, the authors drew the following overall balance: a median AUC with AI of 0.72 (0.62–0.90) compared with a value of 0.61 (0.54–0.69) for the combination AI + clinical risk tools.

Diaz’s overview [22], without a systematic literature research, provided the reader with an overview of the state-of-the-art, dividing the primary studies based on the AI application strategies: (i) used AI as concurrent decision support; (ii) used AI as an independent standalone second reader of screening; (iii) used AI as a triage tool, low risk exams were single read and high-risk exams were double read; and (iv) used AI as a triage tool, where low risk exams were automatically labeled as normal and high-risk exams were double read.

### 3.3. Primary Studies

The common feature of primary studies concerned the type of study: they were in fact all retrospective studies, with the exception of only one randomized trial [23] and one prospective study [24]. However, the outcomes of retrospective studies were different, as they did not allow the overall results to be summarized in a quantitative manner. Furthermore, as highlighted in Figure 2, the studies differed mainly in the procedure with which the AI was applied. In fact, we could consider the following methods: (i) effectiveness studies in which AI was used in the context of retrospective data to analyze its diagnostic capacity without a human reader; (ii) effectiveness studies in which the diagnostic capacity of AI was compared with other clinically diagnostic tools that validated the risk of malignant neoplasm; (iii) comparison studies in which the diagnostic efficacy was compared with the human reader such as double reading vs. single reading + AI; and (iv) effectiveness studies between different software AI and human reading approaches (Table 2).

Regarding the AI programs, Transpara, MIRAI, LUNIT, ResNet, and others were used.

Finally, concerning the origin of the data, 14 out of 26 studies used histopathology data from the Cancer Registry.

### 3.4. RTC and Prospective Studies

In the trial conducted in Sweden by Lang et al. [23], 80,033 women aged between 40 and 80 years were enrolled through organized screening and randomly assigned either to the classic diagnosis method with double reading or to the single reading aided by AI. Cancer detection rates were 6.1 (95% CI 5.4–6.9) per 1000 screened participants in the intervention group, above the lowest acceptable limit for safety, and 5.1 (4.4–5.8) per 1000 in the control group with a ratio of 1.2 (95% CI 1.0–1.5; *p* = 0.052). Recall rates were 2.2% (95% CI 2.0–2.3) in the intervention group and 2.0% (1.9–2.2) in the control group. The false-positive rate was 1.5% (95% CI 1.4–1.7) in both groups. The PPV of recall was 28.3% (95% CI 25.3–31.5) in the intervention group and 24.8% (21.9–28.0) in the control group. In the intervention group, 184 (75%) out of 244 cancers detected were invasive and 60 (25%) were in situ; in the control group, 165 (81%) out of 203 cancers were invasive and 38 (19%) were in situ. The screen-reading workload was reduced by 44.3% using AI.

Demrower’s prospective study concerned women participating in organized population-based screening [24]. The women underwent mammography with two reading modes: traditional double reading and single reader + AI. The following results were obtained: AI was non-inferior for cancer detection compared with double reading by two radiologists, 261 (0.5%) vs. 250 (0.4%) detected cases with a relative proportion of 1.04 (95% CI; 1.00–1.09); single reading by AI with 246 (0.4%) vs. 250 (0.4%) detected cases and a relative proportion 0.98 (95% CI; 0.93–1.04); triple reading by two radiologists + AI with 269 (0.5%) vs. 250 (0.4%) detected cases and a relative proportion of 1.08 (95% CI; 1.04–1.11) were also non-inferior to double reading by two radiologists [24].

1. Retrospective studies from organized screening programs

Seven studies reported data from European countries offering organized screening programs: Hungary [25], UK [25,26], Turkey [27], Norway [28], Denmark [29], Germany [30], Spain [31], Sweden [32], The Netherlands [33], and Switzerland [34]. Of these, three were studies that evaluated the effectiveness of AI tools [28,33], while the remaining seven evaluated the diagnostic effectiveness of AI compared with the reader [25,26,29,30,31,32,33,34].

In the works of Lauritzen and Leibig, the sensitivity of AI was lower than that obtained from the readers, 69.7 vs. 70.8 and 84.6 vs. 87.2, respectively. On the contrary, in Romero Martin and Salim, the sensitivity was greater for AI vs. the readers, 70.8 vs. 63.3 and 86.7 vs. 85, respectively.

With reference to specificity, Lauritzen [29], Leibig [30], and Salim [32] found it to be higher in the readers than in AI: 98.6 vs. 98.8; 93.4 vs. 91.3; 98.5 vs. 92.5, respectively,

It is important to underline that in the works considered, there was no direct comparison between the AUCs obtained with the AI methods and with the traditional double reading method. Hickman’s retrospective study tested the diagnostic performance of three different deep learning models. The diagnostic performance of the DL models was evaluated in two different clinical contexts: in triage, in the identification of suspicious images at time 0, and in the identification of interval cancers. Finally, the DL models were compared with double reading, showing a better sensitivity and a non-inferior specificity for both triage and interval cancer [26].

Furthermore, it is important to highlight some aspects of the studies examined. Sharma’s study was comparative with respect to the use of different mammographs [25]. Beker’s study, conducted on 3228, was different from the previous ones as it compared the AI with only three readers. The AUC of the AI was 0.82 (95% CI, 0.75–0.89) with a sensitivity of 73.7% and specificity of 72%. The AUC of the readers seemed to be lower than the AI, but it was not statistically significant. All readers had higher sensitivity and lower specificity [34].

In the studies in which AI was evaluated as a tool, an AUC of 89.6 was found (Seker’s study) [34]. Larsen’s study, however, focused on the study of the threshold values for identifying a lesion as cancerous, considering a scale from 1 to 5, where one corresponded to an image without suspicion of malignancy and 5 was suspicious of malignancy. The threshold value of 3, 80% of cancers, and 30.7% of interval cancers was observed [28].

Finally, in Wanders’ study, the use of a neural network incorporating both AI and a diagnosis system based on breast density for the diagnosis of interval cancers was evaluated. The results showed how the union of the two methods led to an improvement in diagnosis. However, the data were sensitive to the threshold values that were applied [33].

2. Studies from non-organized screening programs or a sample extracted from organized screening

All studies that used data from non-organized screening or samples selected by the researchers were classified in this category.

In this section, there were twelve studies: three were comparative studies in which the diagnostic performance of the AI was compared with other clinical risk models for breast cancer [35,36,37], and nine in which the AI was used for the diagnostic assessment of radiological images [38,39,40,41,42,43,44,45,46].

Arasu et al. [35] compared AI with a clinical risk tool named the Breast Cancer Surveillance Consortium (BCSC). The comparison highlighted that AI was able to predict the cancer risk better than the clinical risk tool.

Similarly, Lehman’s study tested the application of two risk models, the NCI BCRAT (The Breast Cancer Risk Assessment Tool) and the Tyrer-Cuzick. They essentially showed the diagnostic superiority of artificial intelligence [36].

Similar results were shown by the study by Yala et al., conducted on a larger sample than previous studies [37].

On the other hand, Arefan’s study evaluated the AUC in different mammographic projections compared with breast density, highlighting how the diagnostic performance of mammographic projections was greater than that of breast density [38].

Lang’s study focused on interval cancers, showing how the AI program could act, if correctly programmed, in identifying the tumor and reducing late diagnoses [39].

The studies by Gastoniuoti and Ha highlighted that the CNN (convolutional neural network) model guaranteed better diagnostic performances compared with the image based on breast density alone [40,41].

Hinton’s study recorded a good effectiveness of the deep learning model in correctly identifying tumor images; however, it showed limitations in the identification of interval cancers [42].

Zhu’s study highlighted how a deep learning model, which simultaneously contemplated the radiological image and a clinical risk model, was more effective in the primary diagnosis of cancer, while losing effectiveness in the identification of interval cancers [43]. Sasaki’s study showed a higher AUC in the readers than in AI alone (0.816 vs. 0.706; *p* < 0.001). Similarly, the sensitivity and specificity for the readers were 89% and 86%, respectively, while with AI, establishing cutoffs of 4 and 7, the sensitivity and specificity were 93%, 85%, 45%, and 67%, respectively [44].

Dang’s study, conducted on 314 patients with 12 different radiologists, showed that the AUC improved with the help of AI (0.74 vs. 0.77, *p* = 0.004) [45].

Lee’s study was conducted in South Korea with 200 patients, breast radiologists (BSR), and general radiologists (GR). The AUC of the AI was 0.915 (0.876–0.954), while for radiologists with greater experience in the field of mammography, it was 0.813 (0.756–0.870). The AUC of the inexperienced radiologists in the field of mammography was 0.684 (0.616–0.752). Sensitivity was increased in both groups of radiologists (74.6% vs. 88.6% in BSR, *p* < 0.001; 52.1% vs. 79.4% in GR, *p* < 0.001), while the specificity was not statistically significant (66.6% vs. 66.4% in BSR, *p* = 0.238; 70.8% in GR, *p* = 0.689) [46].

3. Screening from multicenter studies: US, EU, UK, and SWEDEN

Three were multicenter studies and the data came from both organized and non-organized screening programs.

Shaffer’s study involved organized screened patients from Sweden and non-organized programs from the U.S. He highlighted a better performance of AI algorithms in Sweden compared with those in the United States (0.93 vs. 0.858) [47].

McKinney’s study involved the UK (organized screening) and the U.S. (non-organized screening). In the first, the results showed an improvement in specificity of 1.2% compared with the first operator and of 2.7% in sensitivity. Compared with the second reader, however, the AI showed non-inferiority with respect to sensitivity and specificity, as well as for consensus judgment [48].

Kim’s study collected data from South Korea (organized screening) and the U.S. (non-organized screening); the global data highlighted a better performance of the AI vs. the readers (AUC 0.95 vs. 0.81). For this study, no data regarding sensitivity and specificity were available [49].

## 4. Discussion

Artificial intelligence appears to be a promising technology for health, with consequences that can have a major impact on healthcare systems [50]. As reported by Higgins et al., the application of AI may be able to change the approach of doctors and patients to the pathology [51]. The technology appears particularly promising, especially for chronic pathologies in which the clinical history of the disease requires constant monitoring. AI has been applied in the management of chronic lung disease [52,53], renal failure [54], diabetes [55], and ocular pathologies [56]. Oncology represents another potential field of application of AI, particularly linked to image analysis [57,58].

The FDA (Food and Drug Administration) has approved the use of CAD (computer-aided diagnosis) for mammographic images since 1998.

It appears clear that deep learning models that integrate clinical risk scores already validated and in use with image analysis are more effective than individual clinical risk tools based on anamnestic, genetic, anthropometric data and single heart rate analysis and images, highlighting how a multidisciplinary approach is necessary in correct diagnosis. A fundamental point is the number of mammograms performed: increasing the number of exams corresponds to an increase in quality and diagnostic accuracy [59]. This is expressed in the training of AI to search for target images on retrospective data, but above all, when applying AI in the daily reality of screening.

It is important to underline that a large number of mammographic tests occur above all with an organized population-based screening program. In most European countries, this involves double reading and implies: (i) considerable know-how of the diagnostic method; (ii) ad hoc training of the specialists involved; and (iii) the robustness of the organizational infrastructure underlying population-based screening. All the evidence deduced from the literature indicates that it is therefore desirable that all countries equip themselves with a double reading, and that they adopt a universalistic model of the early cancer detection service, which, as demonstrated, is cost effective [60].

If, in the European context, AI does not increase the diagnostic performance, in the American context, where screening is opportunistic and involves only one human reader, the use of AI can increase the diagnostic performance enough to equal that of double human reading. In Denbrowen’s prospective study [24] and Lang’s trial [23], both con-ducted in Sweden, it was highlighted that AI would not actually bring about an improvement in the diagnostic performance already achieved by double human reading. As reported by van Nijnatten TJA, several trials are ongoing, and therefore, there is a growing interest in the use of AI in breast cancer screening [61].

From the analysis of this review, the study of breast density and interval cancer still requires numerous applications. Breast density is an independent risk factor for cancer and has a moderate association with cancer risk, thus requires integration with other diagnostic methods such as ultrasound and magnetic resonance imaging. When appropriately set, AI can be extremely valid in identifying a breast at risk, as shown by the studies by Gastounioti and Ha [40,41].

AI is still not performing well in interval cancers, as seen in the retrospective studies by Hinton [42] and Wanders [33].

Moreover, Combi et al. [62] suggest that an AI system must have the following characteristics: interpretability, understandability, usability, and usefulness. Interpretability is the degree to which the user can understand the ways in which the system makes decisions; understandability is the degree to which the user can understand the result indicated by the system and the mechanism with which the system manages to provide that result; usability concerns the ease of using the interface; and usefulness concerns the usefulness of the system for its intended purpose.

In daily clinical practice, these characteristics could be translated into: (i) a system that is focused on the needs of the doctor, in diagnostic decision making, acting as a natural technological evolution in improving patient care; and (ii) a system that integrates with a patient’s diagnostic and treatment path.

Artificial intelligence (AI) could prove to be a valuable ally in mammographic screening for breast cancer, allowing for the identification of any tumors with the same accuracy as a standard reading performed by two radiologists.

It is necessary to understand whether the integration of the radiologists’ interpretation with AI can help identify those tumors that escape traditional screening because they appear in the interval between one exam and the next as well as the cost-effectiveness of the technology.

If future studies confirm the actual benefit and safety of AI in mammography screening, it could become a support tool to overcome the current shortage of radiologists, at least eliminating the need for double reading or of a third radiologist in the case of disagreement. In this way, specialists could focus on more advanced diagnostics, shortening the waiting times for patients.

In this way, AI does not replace doctors, but helps them make faster and more informed decisions by providing a reliable further opinion.

## 5. Conclusions

However, it should be emphasized that AI systems are often trained on large datasets that can contain biases that reflect historical inequalities or prejudices. Ensuring fairness means recognizing and mitigating bias in data, algorithms, and decision-making processes. AI developers must be mindful of how their systems may unintentionally perpetuate harmful stereotypes or disadvantage certain groups. AI’s ability to collect, analyze, and process vast amounts of personal data raises significant concerns about privacy. Some applications and data mining can lead to invasive surveillance practices. The ethical use of AI involves balancing the benefits of data analysis with the protection of individual privacy rights. Clear guidelines are needed to ensure that personal data are used responsibly and with informed consent.

## Figures and Tables

**Table 1:** Meta-analyses and reviews.

Author	Aims	Patients Population	Results
META-ANALYSIS			
Hickman [19]	Evaluate machine learning (ML) accuracy in detecting breast cancer in screening mammography.	14 eligible studies, 7 triage studies 8 studies on comparison between AI and readers; of these 5 eligible for a meta-analysis 185,252 patients	Triage studies: AI could be used to reduce the number of mammography examinations read by radiologists by 17–91% while “missing” 0–7% of cancers AI vs. readers’ studies AI/ML algorithm Sensitivity 0.75 (0.65–0.83) Specificity 0.90 (0.82–0.95) AUC 0.89 (0.84–0.98) Readers Sensitivity 0.73 (0.61–0.83) Specificity 0.87 (0.72–0.95) AUC 0.85 (0.78–0.97)
Jung Hyun Yoon [20]	A random effects meta-analysis and meta-regression analysis were performed for overall studies and for different study types (reader studies vs. historic cohort studies) and imaging techniques (digital mammography vs. DBT). Conclusions: Standalone AI for screening digital mammography performed as well as or better than the radiologists.	16 studies. 7 historical 6 readers study 4 DBT 1,108,328 mammograms 497,091 women (six reader studies, seven historic cohort studies on digital mammography, and four studies on digital breast tomography (DBT).	AI standalone AUCs were significantly higher for standalone AI than radiologists in the six reader studies on digital mammography (0.87 vs. 0.81, *p* = 0.002), but not for historic cohort studies (0.89 vs. 0.96, *p* = 0.152). Four studies on DBT showed significantly higher AUCs in AI compared with radiologists (0.90 vs. 0.79, *p* < 0.001). Higher sensitivity and lower specificity were seen for standalone AI compared with radiologists.
REVIEW AND SYSTEMATIC REVIEW			
Schopf [21]	Summarize the literature regarding the performance of mammography-image based artificial intelligence (AI) algorithms, with and without additional clinical data, for future breast cancer risk prediction.	16 studies	The median AUC performance of AI image-only models was 0.72 (range 0.62–0.90) compared with 0.61 for breast density or clinical risk factor-based tools (range 0.54–0.69). Of the seven studies that compared AI image-only performance directly to combined image + clinical risk factor performance, six demonstrated no significant improvement, and one study demonstrated increased improvement.
Diaz [22]	Overview of the current state of artificial intelligence (AI) technology for automated detection of breast cancer in digital mammography (DM) and digital breast tomosynthesis (DBT). Aimed to discuss the technology, available AI systems, and the challenges faced by AI in breast cancer screening.		DL-based AI systems have shown significant improvements in breast cancer detection. They have the potential to enhance screening outcomes, reduce false negatives and positives, and detect subtle abnormalities missed by human observers. However, challenges like the lack of standardized datasets, potential bias in training data, and regulatory approval hinder their widespread adoption.

Abbreviations: AI, artificial intelligence; DL, deep learning; SE, sensibility; SP, specificity; BDT, digital breast tomography; DR, double reading.

**Table 2:** Studies reporting data on AI performance according to patient recruitment.

RECRUIMENT: ORGANIZED SCREENING							
Author, Country, Study Publication Year	Study Design	Outcomes	Patient Population				Outcome Results
			Period of Collection Mammograms and Woman’s Age	No. of Mammograms/Patients	Mammography Reading Protocol and AI Reading Protocol	Diagnosis Confirmations	
Lång Sweden [23]	RCT Comparing study	Cancer detection rate Recall rates Workload reduction	12 April 2021 28 July 2022 40–74 years	80,033 AI-supported screening (*n* = 40,003) or double reading without AI (*n* = 40,030)	DR Transpara		Cancer detection rates were 6.1 (95% CI 5.4–6.9) per 1000 screened participants in the intervention group, and 5.1 (4.4–5.8) per 1000 in the control group-a ratio of 1.2 (95% CI 1.0–1.5; *p* = 0·052). Recall rates were 2.2% (95% CI 2.0–2.3) in the intervention group and 2.0% (1.9–2.2) in the control group. The false-positive rate was 1.5% (95% CI 1.4–1.7) in both groups. The PPV of recall was 28.3% (95% CI 25.3–31.5) in the intervention group and 24.8% (21.9–28.0) in the control group. The screen-reading workload was reduced by 44.3% using AI.
Dembrower Sweden [24]	Prospective study Comparing study	Cancer detection rate in single reading with AI, double reading with AI, and triple reading with AI	1 April 2021 9 June 2022 40–74 years	58,344			Double reading by one radiologist plus AI was non-inferior for cancer detection compared with double reading by two radiologists (261 [0.5%] vs. 250 [0.4%] detected cases relative proportion 1.04 [95% CI 1.00–1.09]). Single reading by AI (246 [0.4%] vs. 250 [0.4%] detected cases; relative proportion 0.98 [0.93–1.04]) Triple reading by two radiologists plus AI (269 [0.5%] vs. 250 [0.4%] detected cases; relative proportion 1.08 [1.04–1.11]) were also non-inferior to double reading by two radiologists.
Sharma Hungary and UK 2023 [25]	Retrospective Comparing study	SE, SP, PPV Recall rate workload for each mammography equipment	2009–2012 50–70 years	304,360/-	Double reading Mia version 2.0	Histopathology (Hungarian data) Cancer Registry (UK data)	DR with AI, compared with human DR, showed at least a non-inferior recall rate, cancer detection rate, sensitivity, specificity and positive predictive value (PPV) for each mammography vendor and site, and superior recall rate, specificity, and PPV for two systems. The simulation indicates that using AI would have increased the arbitration rate (3.3% to 12.3%) but could have reduced the human workload by 30.0% to 44.8%.
Hickman UK 2023 [26]	Retrospective AI tool efficacy study	Three different DL models as triage and in interval cancers in a possible second automatized second reading	January 2017 to December 2018	78,849	CAD, Deep learning	Histopathology	Rule-out triage: Models DL-1, DL-2, and DL-3 triaged 35.0% (27,565 of 78,849), 53.2% (41,937 of 78,849), and 55.6% (43,869 of 78,849) of mammograms, respectively, with 0.0% (0 of 887) to 0.1% (one of 887) of the screening-detected cancers undetected. Interval cancers: DL algorithms triaged in 4.6% (20 of 439) to 8.2% (36 of 439) of interval and 5.2% (36 of 688) to 6.1% (42 of 688) of subsequent-round cancers when applied after the routine double-reading workflow. Both approaches: overall noninferior specificity (difference, −0.9%; *p* < 0.001) and superior sensitivity (difference, 2.7%; *p* < 0.001) for the adaptive workflow compared with routine double reading for all three algorithm
Seker Turkey 2024 [27]	Retrospective AI tool efficacy study	SE, SP, AUC	2009 to 2019	22,621/8758 Woman’s age: Not reported	Double reading Lunit INSIGHT MMG version 1.1.7.1 Positive BIRADS 0, 3, 4, 5 Negative 1–2	Not reported	AUC: 89.6 (86.1–93.2%) SE 72.38 SP 92.86
Larsen Norway 2022 [28]	Retrospective AI tool efficacy study	Rate of cancer detection	2009–2018 Woman’s age: 50–69 years	22,969/ 478,772	Transpara 1.7 1–5 to indicate suspicion of malignancy—1–2 negative, probably benign; 3–5 from suspicion of malignancy to malignancy	Cancer Registry	A total of 653/752 screen-detected cancers (86.8%) and 92/205 interval cancers (44.9%) were given a score of 10 by the AI system (threshold 1). Using a threshold of 3, 80.1% of the screen-detected cancers (602/752) and 30.7% of the interval cancers (63/205) were selected. Screen-detected cancers with AI scores not selected using the thresholds had favorable histopathologic characteristics compared with those selected; contrasting results were observed for interval cancer.
Lauritzen Denmark 2022 [29]	Retrospective Comparing study	AUC, SE, SP of AI versus readers’ performance for AI performance according to BI-RADS for all cancers, screen detected, interval cancers, and long-term cancers	2012–2013 Woman’s age: 50–69 years	54,977	Transpara 1.7 BI-RADS	Histopathology	AI AUC 0.97 (0.97–0.98) SE 69.7 (66.9–72.4) SP 98.6 (98.5–98.7) Readers AUC not rep. SE 70.8 (68.0–73.5) SP 98.6 (98.5–98.7)
Leibig Germany 2022 [30]	Retrospective Comparing study	AUC, SE, SP of AI versus readers’ performance	2007–2020 Woman’s age: 50–70 years	1,193,197		Histopathology	AI AUC 0.94 (0.939–0.950) SE 84.6 (83.5–85.9) SP 91.3 (91.1–91.5) Readers AUC not rep. SE87.2 (58.2–75.2) SP 93.4 (93.2–93.6)
Romero Martin Spain 2022 [31]	Retrospective Comparing study	AUC, SE, SP of AI versus	2015–2016 Woman’s age: 50–69	15,999	Transpara 1.7 BI-RADS	Histopathology	AI AUC 0.94 (0.91–0.97) SE 70.8 (61.8–78.4) SP NR Readers AUC not rep. SE 67.3 (58.2–75.2) SP NR
Salim Sweden 2020 [32]	Retrospective Comparing study	AUC, SE, SP of 3 different AI; Overall, SE, SP of AI; readers’ SE, SP	2008/2015 Woman’s age: 50–69	8805	2 readers; 25 different; first radiologist for first reading and 20 for second reading CAD based	Cancer Registry	AUC AI-1 0.956 (95% CI, 0.948–0.965), SE 81.9; sp: 96.1 AI-2 0.922 (95% CI, 0.910–0.934) SE 67.0; SP: 96.6 AI-3 0.920 (95% CI, 0.909–0.931) SE 67.4; SP: 96.7 Overall AI: SE 86.7% (95% CI, 84.2–89.2%) and specificity of 92.5% (95% CI, 92.3–92.7%) Overall readers: SP was 98.5 (98.4–98.6) and SE 85.0 (82.2–87.5)
Wanders The Netherlands 2022 [33]	Retrospective nested case–control AI tool efficacy study	Interval cancer (IC) risk prediction	January 2011 January 2015 Woman’s age: according to organized screening	1,163,147	Neural network (NN)-based model	Cancer Registry	AUC of the NN model was 0.79 (95% CI: 0.77, 0.81), which was higher than the AUC of the AI cancer detection system or breast density alone (AUC, 0.73 [95% CI: 0.71, 0.76] and 0.69 [95% CI: 0.67, 0.71], respectively; *p* < 0.001 for both). At 90% specificity, the NN model had a sensitivity of 50.9% (339 of 666 women; 95% CI: 45.2, 56.3) for the prediction of IC, which was higher than that of the AI system (37.5%; 250 of 666 women; 95% CI: 33.0, 43.7; *p* < 0.001) or breast density percentage alone (22.4%; 149 of 666 women; 95% CI: 17.9, 28.5; *p* < 0.001).
Beker Switzerland 2017 [34]	Retrospective Comparing study	AUC, SE, SP of AI versus AUC, SE, SP of 3 different readers	2012	-/3228	ViDi Suite Version 2.0; ViDi Systems Inc, Villaz-Saint-Pierre, Switzerland) to 3 radiologist s 7, 10, and 3 years of experience in breast imaging BI-RADS	Histopathology	AUC of AI of 0.82 (95% CI, 0.75–0.89) with an optimal sensitivity/specificity of 73.7/72.0%. Diagnostic accuracy measured by AUC was not significantly different between the readers (AUC = 0.79, 0.77, and 0.87; *p* = 0.18, 0.32, and 0.83) or the AI (*p* = 0.45, 0.56, and 0.62). However, all readers exhibited a higher specificity but lower sensitivity when compared with the ANN, with a sensitivity/specificity of 60.0%/94.4% for reader 1, 60.0%/93.6% for reader 2, and 80.0%/90.2% for reader 3.
**RECRUIMENT: Studies from Non-Organized Screening Programs or a Sample Came from Organized Screening**							
**Author, Country,** **Study Publication Year**	**Study Design**	**Outcomes**	**Patient Population**				**Outcome Results**
			**Period of Collection Mammograms and Woman Age**	**No. of Mammograms/Patients**	**Mammography Reading Protocol and AI Reading Protocol**	**Diagnosis Confirmations**	
Arasu USA 2023 [35]	Retrospective, case–control AI tools study	Prediction of 5-year risk between AI tools and AI tool and Breast Cancer Surveillance Consortium (BCSC)	2016 and 2021	13,628	Mirai Globally-Aware Multiple Instance Classifier MammoScreen ProFound AI and Mia	Kaiser Permanente Northern California Breast Cancer Tracking System	AI predicted incident cancers at 0 to 5 years better than the Breast Cancer Surveillance Consortium (BCSC) clinical risk model (AI time-dependent area under the receiver operating characteristic curve [AUC] range, 0.63–0.67; BCSC time-dependent AUC, 0.61; Bonferroni-adjusted *p* < 0.0016). Combining AI algorithms with BCSC slightly improved the time-dependent AUC versus AI alone (AI with BCSC time-dependent AUC range, 0.66–0.68; Bonferroni-adjusted *p* < 0.0016).
Lehman USA 2022 [36]	Retrospective, case–control AI tools study	AI detecting cancer vs. a NCI BCRAT risk model from 18 September 2017 to 1 February 2021	From 18 September 2017 to 1 February 2021	57,635 consecutive patients with a prior mammogram underwent 119,179 bilateral screening mammograms.	Deep learning	Not reported	Cancers detected per thousand patients screened were higher in patients at increased risk by the deep learning model (8.6, 95% confidence interval [CI] = 7.9 to 9.4) compared with the Tyrer-Cuzick (4.4, 95% CI = 3.9 to 4.9) and NCI BCRAT (3.8, 95% CI = 3.3 to 4.3) models (*p* < 0.001). Area under the receiver operating characteristic curves of the deep learning model (0.68, 95% CI = 0.66 to 0.70) was higher compared with the Tyrer-Cuzick (0.57, 95% CI = 0.54 to 0.60) and NCI BCRAT (0.57, 95% CI = 0.54 to 0.60) models. Simulated screening of the top 50th percentile risk by the deep learning model captured statistically significantly more patients with cancer compared with Tyrer-Cuzick and NCI BCRAT models (*p* < 0.001).
Yala USA 2022 [37]	Retrospective, case–control AI tools study	Deep learning (DL) vs. cancer risk model	1 January 2009, and 31 December 2012	88,994 consecutive screening mammograms in 39,571 women	Deep learning (DL)	Not reported	The test set included 3937 women, aged 56.20 years ± 10.04. Hybrid DL and image-only DL showed AUCs of 0.70 (95% confidence interval [CI]: 0.66, 0.75) and 0.68 (95% CI: 0.64, 0.73), respectively. RF-LR and TC showed AUCs of 0.67 (95% CI: 0.62, 0.72) and 0.62 (95% CI: 0.57, 0.66), respectively. Hybrid DL showed a significantly higher AUC (0.70) than TC (0.62; *p* < 0.001) and RF-LR (0.67; *p* = 0.01).
Arefan USA 2020 [38]	Retrospective, case–control AI tools study	AUC between different mammography projections	January 2007 and January 2012	226	Deep learning model and a GoogLeNet-LDA	Not reported	AUC was 0.73 (95% Confidence Interval [CI]: 0.68–0.78; GoogLeNet-LDA model on CC view) when using the whole breast was 0.72 (95% CI: 0.67–0.76; GoogLeNet-LDA model on MLO + CC view) when using the dense tissue, respectively, as the model input. The GoogLeNet-LDA model significantly (all *p* < 0.05) outperformed the end-to-end GoogLeNet model in all experiments. CC view was consistently more predictive than MLO view in both deep learning models, regardless of the input sub-regions. Both models exhibited superior performance than the percent breast density (AUC = 0.54; 95% CI: 0.49–0.59).
Lang Sweden 2021 [39]	Retrospective, case–control AI tools study	Preceding screening mammograms of cancer in southern Sweden were analyzed with a deep learning-based AI system	2013 and 2017	429 consecutive women diagnosed with interval	Not reported	Not reported	A statistically significant correlation between the interval cancer classification groups and AI risk score was observed (*p* < 0.0001). AI scored one in three (143/429) interval cancers with a risk score of 10, of which 67% (96/143) were either classified as minimal signs or false negatives. Of these, 58% (83/143) were correctly located by AI and could therefore potentially be detected at screening with the aid of AI, resulting in a 19.3% (95% CI 15.9–23.4) reduction of interval cancers. At the 4% and 1% recall thresholds, the reduction in interval cancers was 11.2% (95% CI 8.5–14.5) and 4.7% (95% CI 3.0–7.1). The corresponding reduction in interval cancers with grave outcomes (women who died or with stage IV disease) at a risk score of 10 was 23% (8/35; 95% CI 12–39).
Gastounioti USA 2018 [40]	Retrospective, case–control AI tools study	Breast density	2002–2006	5139 176 cases and 4963 controls	Convolutional neural network	Not reported	Strong linear separability of cancer cases from the controls was demonstrated on the basis of the five meta-features generated by the proposed hybrid framework. The corresponding case–control classification performance was AUC = 0.90 (95% CI: 0.82–0.98), with a sensitivity and specificity equal to 0.81 and 0.98, respectively.
Ha USA 2019 [41]	Retrospective, case–control AI tools study	Breast density	January 2011 to January 2017	1474 mammographic images First, 210 patients with new incidence of breast cancer were identified. The control group consisted of 527 patients without breast cancer from the same time period	Convolutional neural network	Not reported	Breast density (BD) was significantly higher in the case group [2.39 (SD, 0.7)] than the control group [1.98 (SD, 0.75), *p* < 0.0001]. In multivariate logistic regression analysis, both the CNN pixel-wise mammographic risk model and BD were significant independent predictors of breast cancer risk (*p* < 0.0001). The CNN risk model showed greater predictive potential [OR = 4.42 (95% CI, 3.4–5.7)] compared with BD [OR = 1.67 (95% CI, 1.4–1.9)].
Hinton USA 2019 [42]	Retrospective, case–control AI tools study	Interval cancer	2006 and 2015	A total of 316,001 examinations were performed in the screening population, leading to a total of 245 interval cancers of which 182 women were available for this study	ResNet-50	Not reported	The optimized deep learning model achieved an AUC of 0.82. Contingency table analysis showed the network was correctly classifying 75.2% of the mammograms and that incorrect classifications were slightly more common for the interval cancer mammograms.
Zhu USA 2021 [43]	Retrospective, case–control AI tools study	Ability of DL models to estimate the risk of interval and screening-detected breast cancers with and without clinical risk factors	January 2006 December 2013.	25,096 digital screening mammograms	Deep learning	Not reported	Cancer diagnosis DL model: The C statistics and odds ratios for comparing patients with screening-detected cancer versus the matched controls were 0.66 (95% CI: 0.63, 0.69) and 1.25 (95% CI: 1.17, 1.33). Clinical risk factors with the Breast Imaging Reporting and Data System (BI-RADS) density model: 0.62 (95% CI: 0.59, 0.65) and 2.14 (95% CI: 1.32, 3.45). Combined DL and clinical risk factors model 0.66 (95% CI: 0.63, 0.69) and 1.21 (95% CI: 1.13, 1.30) Interval cancer DL model: For comparing patients with interval cancer versus controls, the C statistics and odds ratios were 0.64 (95% CI: 0.58, 0.71) and 1.26 (95% CI: 1.10, 1.45), The risk factors with BI-RADS density: 0.71 (95% CI: 0.65, 0.77) and 7.25 (95% CI: 2.94, 17.9) Combined DL and clinical risk factors model: 0.72 (95% CI: 0.66, 0.78) and 1.10 (95% CI: 0.94, 1.29) for the The *p* values between the DL, BI-RADS, and combined model’s ability to detect screen and interval cancer were 0.99, 0.002, and 0.03, respectively.
Sasaki Japan 2020 [44]	Retrospective Comparing study	AUC, SE, SP of 3 different AIs; Overall, SE, SP of AI; Readers SE, SP	January 2018 and October 2018	310	Transpara	Not reported	The AUC was higher for human readers than with the standalone Transpara system (human readers 0.816; Transpara system 0.706; difference 0.11; *p* < 0.001). The sensitivity of the unaided HR for diagnosis was 89% and the specificity was 86%. The sensitivity of the standalone Transpara system for cutoff scores of 4 and 7 were 93% and 85%, and the specificities were 45% and 67%, respectively.
Dang France 2022 [45]	Retrospective Comparing study	AUC, SE, SP of 3 different AIs; Overall, SE, SP of AI; Readers SE, SP	June 2012 to March 2020	314	Mammoscreen forced BI-RADS score of 1–5 per breast	Histopathology	AUC was significantly improved when using AI (0.74 vs. 0.77, *p* = 0.004).
Lee Korea 2022 [46]	Retrospective Comparing study	AUC, SE, SP of 3 different AIs; Overall, SE, SP of AI; Readers SE, SP, according to their experience in the field (breast radiology expertise vs. general radiologist)	March 2009 and September 2018	200	Lunit INSIGHT MMG, version 1.1.1.0; Lunit Scale from 1 (normal) to 7 (highly suggestive of malignancy)	Histopathology	The AUROC of the AI alone, BSR (average across five readers), and GR (average across five readers) groups was 0.915 (95% c. i., 0.876–0.954), 0.813 (0.756–0.870), and 0.684 (0.616–0.752), respectively. With AI assistance, the AUROC significantly increased to 0.884 (0.840–0.928) and 0.833 (0.779–0.887) in the BSR and GR groups, respectively (*p* = 0.007 and *p* < 0.001, respectively). Sensitivity was improved by AI assistance in both groups (74.6% vs. 88.6% in BSR, *p* < 0.001; 52.1% vs. 79.4% in GR, *p* < 0.001), but the specificity did not differ significantly (66.6% vs. 66.4% in BSR, *p* = 0.238; 70.8% vs. 70.0% in GR, *p* = 0.689).
**RECRUIMENT: Screening from Multicenter Studies: US, EU, UK, and SWEDEN**							
**Author, Country,** **Study Publication Year**	**Study Design**	**Outcomes**	**Patient Population**				**Outcome Results**
			**Period of Collection Mammograms and Woman Age**	**No. of Mammograms/Patients**	**Mammography Reading Protocol and AI Reading Protocol**	**Diagnosis Confirmations**	
Schaffer Sweden and USA 2020 [47]	Retrospective Comparing study	Outcomes: AUC, SE, SP of AI versus AUC, SE, SP of AI of Swedish court and American readers	From April 2008 to December 2012	2	Different protocols, according to aims of the study	Histopathology	The top-performing algorithm achieved an area under the curve of 0.858 (United States) and 0.903 (Sweden) and 66.2% (United States) and 81.2% (Sweden) specificity at the radiologists’ sensitivity, lower than the community-practice radiologists’ specificity of 90.5% (United States) and 98.5% (Sweden). Combining top-performing algorithms and U.S. radiologist assessments resulted in a higher area under the curve of 0.942 and achieved a significantly improved specificity (92.0%) at the same sensitivity.
McKinney, UK and USA 2020 [48]	Retrospective Comparing study	Outcomes: % improving of SE, SP between first and second readers with AI in UK; % improving of SE, SP with AI in USA	2001 and 2018	25,856	Two readers in UK, one reader in the USA In the UK, two readers, and in cases of disagreement, an arbitration process could invoke a third opinion. In the USA, each mammogram was interpreted by a single radiologist. BI-RADS	Biopsy	Compared with the first reader, the AI system demonstrated an improvement in specificity of 1.2% (95% C.I. 0.29%, 2.1%; *p* = 0.0096 for superiority) and an improvement in sensitivity of 2.7% (95% C.I. 3%, 8.5%; *p* = 0.004). Compared with the second reader, the AI system showed non-inferiority (at a 5% margin) for both specificity (*p* < 0.001) and sensitivity (*p* = 0.02). Likewise, the AI system showed non-inferiority (at a 5% margin) to the consensus judgment for the specificity (*p* < 0.001) and sensitivity (*p* = 0.0039). Compared with the typical reader, the AI system demonstrated an improvement in specificity of 5.7%.
Kim South Korea and USA 2020 [49]	Retrospective Comparing study	Outcomes: AUC of AI; versus AUC of readers even according to BI-RADS category	January 2004–December 2016, in South Korea; January 2000–December 2018, in the USA; and January 2010–December 2018, in the UK	166,578/68,008	ResNet BI-RADS (four category)	Histopathology	AI AUC 0.95 (0.93–0.96) SE NR SP NR Readers AUC 0.81 (0.77–0.85) SE NR SP NR

Abbreviations: AI artificial intelligence; DL deep learning; SE sensibility; SP specificity; PPV, positive predict value; NPV negative predict value; NR not reported; CI confidence interval; DR double reading.

## Supplementary Materials

The following supporting information can be downloaded at: https://www.mdpi.com/article/10.3390/healthcare13040378/s1, Table S1. Search strategy; Table S2. Risk of Bias according to AMSTAR 2 scale; Table S3. Risk of Bias for RTC according to Cochrane Collaboration; Table S4. Risk of bias of Cohort Studies according to New Castle Ottawa scale; Table S5. Preferred Reporting Items for Systematic reviews and Meta-Analyses extension for Scoping Reviews (PRISMA-ScR) Checklist.
