# Supplementary material for: The Future of Breast Cancer Organized Screening Program Through Artificial Intelligence: A Scoping Review
Source: Healthcare (Basel). 2025 Feb 10;13(4):378. doi: 10.3390/healthcare13040378 (PMC11855082; doi:10.3390/healthcare13040378)
Supplement: Supplementary file 1 [file healthcare-13-00378-s001.zip › healthcare-3433662-supplementary.pdf]

Supplementary materials

Table S1. Search strategy

Table S2. Risk of Bias according to AMSTAR 2 scale

Table S3. Risk of Bias for RTC according to Cochrane Collaboration

Table S4. Risk of bias of Cohort Studies according to New Castle Ottawa scale.

Table S5. Preferred Reporting Items for Systematic reviews and Meta-Analyses extension for Scoping Reviews (PRISMA-ScR) Checklist

Table S1. Search strategy

**PUBMED**	
**(artificial intelligence) AND ((breast cancer screening) AND (mammography))** Filters: **in the last 10 years, English, Female**<br>(("artificial intelligence"[MeSH Terms] OR ("artificial"[All Fields] AND "intelligence"[All Fields]) OR "artificial intelligence"[All Fields]) AND (("breast neoplasms"[MeSH Terms] OR ("breast"[All Fields] AND "neoplasms"[All Fields]) OR "breast neoplasms"[All Fields] OR ("breast"[All Fields] AND "cancer"[All Fields]) OR "breast cancer"[All Fields]) AND ("diagnosis"[MeSH Subheading] OR "diagnosis"[All Fields] OR "screening"[All Fields] OR "mass screening"[MeSH Terms] OR ("mass"[All Fields] AND "screening"[All Fields]) OR "mass screening"[All Fields] OR "early detection of cancer"[MeSH Terms] OR ("early"[All Fields] AND "detection"[All Fields] AND "cancer"[All Fields]) OR "early detection of cancer"[All Fields] OR "screen"[All Fields] OR "screenings"[All Fields] OR "screened"[All Fields] OR "screens"[All Fields]) AND ("mammography"[MeSH Terms] OR "mammography"[All Fields] OR "mammographies"[All Fields] OR "mammography s"[All Fields]))) AND ((y_10[Filter]) AND (female[Filter]) AND (english[Filter]))	659
**EMBASE**	

('artificial intelligence'/exp OR 'artificial intelligence') AND ('mammography'/exp OR mammography) AND ('breast cancer'/exp OR 'breast cancer' OR (('breast'/exp OR breast) AND ('cancer'/exp OR cancer))) AND ('early detection'/exp OR 'early detection' OR (early AND ('detection'/exp OR detection)))	195
**SCOPUS**	
artificial AND intelligence AND mammography AND breast AND cancer AND screening AND early AND detection	280
WEB OF SCIENCE	119
breast cancer and intelligence artificial and screening and AUC	

Table S2. Risk of Bias according to AMSTAR 2 scale

Items	Hickman [19]	Jung Hyun Yoon [20]	Schopf [21]	Diaz [22]
Did the research questions and inclusion criteria for the review include the components of PICO?	YES	YES	YES	YES
Did the report of the review contain an explicit statement that the review methods were established prior to the conduct of the review and did the report justify any significant deviations from the protocol?	YES	YES	YES	YES
Did the review authors explain their selection of the study designs for inclusion in the review?	YES	YES	YES	YES
Did the review authors use a comprehensive literature search strategy?	YES	YES	YES	YES
Did the review authors perform study selection in duplicate?	YES	YES	YES	YES
Did the review authors perform data extraction in duplicate?	YES	YES	YES	YES
Did the review authors provide a list of excluded studies and justify the exclusions?	YES	YES	YES	YES
Did the review authors describe the included studies in adequate detail?	YES	YES	YES	YES
Did the review authors use a satisfactory technique for assessing the risk of bias (RoB) in individual studies that were included in the review?	YES	YES	YES	YES
Did the review authors report on the sources of funding for the studies included in the review?	NO	NO	NO	NO
If meta-analysis was performed did the review authors use appropriate methods for statistical combination of results?	YES	YES	NOT APPLICABLE	NOT APPLICABLE
If meta-analysis was performed, did the review authors assess the potential impact of RoB in individual studies on the results of the meta-analysis or other evidence synthesis?	NO	NO	NOT APPLICABLE	NOT APPLICABLE

Did the review authors account for RoB in individual studies when interpreting/ discussing the results of the review?	NO	NO	NO	NO
Did the review authors provide a satisfactory explanation for, and discussion of, any heterogeneity observed in the results of the review?	NO	NO	NOT APPLICABLE	NOT APPLICABLE
Did the review authors provide a satisfactory explanation for, and discussion of, any heterogeneity observed in the results of the review?	NO	NO	NOT APPLICABLE	NOT APPLICABLE
If they performed quantitative synthesis did the review authors carry out an adequate investigation of publication bias (small study bias) and discuss its likely impact on the results of the review?	NO	NO	NOT APPLICABLE	NOT APPLICABLE
Did the review authors report any potential sources of conflict of interest, including any funding they received for conducting the review?	NO	NO	NOT APPLICABLE	NOT APPLICABLE

Table S3. Risk of Bias for RTC according to Cochrane Collaboration

	**Selection bias**		**Detection bias**	**Attrition bias**
Study	**Random sequence generation**	**Allocation concealment**	**Blinding**	**Incomplete outcome data**
**Lång**	Low	Low	Low	Low

Table S4. Risk of bias of Cohort Studies according to New Castle Ottawa scale.

	**Selection**				**Comparability**	**Outcome**		
**Study**	**Representativeness of the exposed cohort**	**Selection of the non exposed cohort**	**Ascertainment of exposure**	**Demonstration that outcome of interest was not present at start of study**	Comparability of cohorts on the basis of the design or analysis	Assessment of outcome	Was follow-up long enough for outcomes to occur	Adequacy of follow up of cohorts
Dembrower	A	A	B	A	A	B	A	A
Sharma	A	A	B	B	A	B	A	A
Hickman	A	A	B	B	A	B	A	A
Seker	A	A	B	B	A	B	A	A
Larsen	A	A	B	B	A	B	A	A

Lauritzen	A	A	B	B	A	B	A	A
Leibig	A	A	B	B	A	B	A	A
Romero Martin	A	A	B	B	A	B	A	A
Salim	A	A	B	B	A	B	A	A
Wanders	A	A	B	B	A	B	A	A
Beker	A	A	B	B	A	B	A	A
Arasu	B	B	B	B	A	B	B	A
Lehman	B	B	B	B	A	B	B	A
Yala	B	B	B	B	A	B	B	A
Arefan	B	B	B	B	A	B	B	A
Lang	B	B	B	B	A	B	B	A
Gastouni oti	B	B	B	B	A	B	B	A
Ha	B	B	B	B	A	B	B	A
Hinton	B	B	B	B	A	B	B	A
Zhu	B	B	B	B	A	B	B	A
Sasaki	B	B	B	B	A	B	B	A
Dang	B	B	B	B	A	B	B	A
Lee	B	B	B	B	A	B	B	A
Schaffer	A	A	B	B	A	B	A	A
McKinne y	A	A	B	B	A	B	A	A
Kim	A	A	B	B	A	B	A	A

Selection

1) <u>Representativeness of the exposed cohort</u>

a) truly representative of the average of breast cancer in the community

b) somewhat representative of the average of breast cancer in the community

c) selected group of users eg nurses, volunteers

d) no description of the derivation of the cohort

2) Selection of the non exposed cohort

a) drawn from the same community as the exposed cohort

b) drawn from a different source

c) no description of the derivation of the non exposed cohort

3) Ascertainment of exposure

a) secure record (eg surgical records)

b) structured interview

c) written self report

d) no description

4) Demonstration that outcome of interest was not present at start of study

a) yes

b) no

Comparability

1) Comparability of cohorts on the basis of the design or analysis

a) study controls for ⚜

b) study controls for any additional.

Outcome

1) Assessment of outcome

a) independent blind assessment

b) record linkage

c) self report

d) no description

2) Was follow-up long enough for outcomes to occur

a) yes (select an adequate follow up period for outcome of interest)

b) no

3) Adequacy of follow up of cohorts

a) complete follow up - all subjects accounted for

b) subjects lost to follow up unlikely to introduce bias - small number lost - > 3% follow up, or description provided of those lost) ⚜

c) follow up rate < 80% and no description of those lost

d) no statement

Table S5. Preferred Reporting Items for Systematic reviews and Meta-Analyses extension for Scoping Reviews (PRISMA-ScR) Checklist

SECTION	ITEM	PRISMA-ScR CHECKLIST ITEM	REPORTED ON PAGE #
**TITLE**			
Title	1	Identify the report as a scoping review.	1
**ABSTRACT**			

SECTION	ITEM	PRISMA-ScR CHECKLIST ITEM	REPORTED ON PAGE #
Structured summary	2	Provide a structured summary that includes (as applicable): background, objectives, eligibility criteria, sources of evidence, charting methods, results, and conclusions that relate to the review questions and objectives.	2
**INTRODUCTION**			
Rationale	3	Describe the rationale for the review in the context of what is already known. Explain why the review questions/objectives lend themselves to a scoping review approach.	3
Objectives	4	Provide an explicit statement of the questions and objectives being addressed with reference to their key elements (e.g., population or participants, concepts, and context) or other relevant key elements used to conceptualize the review questions and/or objectives.	3
**METHODS**			
Protocol and registration	5	Indicate whether a review protocol exists; state if and where it can be accessed (e.g., a Web address); and if available, provide registration information, including the registration number.	Not applicable
Eligibility criteria	6	Specify characteristics of the sources of evidence used as eligibility criteria (e.g., years considered, language, and publication status), and provide a rationale.	3,4, supplementary table 1
Information sources*	7	Describe all information sources in the search (e.g., databases with dates of coverage and contact with authors to identify additional sources), as well as the date the most recent search was executed.	3,4
Search	8	Present the full electronic search strategy for at least 1 database, including any limits used, such that it could be repeated.	3,4, supplementary table 1
Selection of sources of evidence†	9	State the process for selecting sources of evidence (i.e., screening and eligibility) included in the scoping review.	3,4, supplementary table 1
Data charting process‡	10	Describe the methods of charting data from the included sources of evidence (e.g., calibrated forms or forms that have been tested by the team before their use, and whether data charting was done independently or in duplicate) and any processes for obtaining and confirming data from investigators.	3,4
Data items	11	List and define all variables for which data were sought and any assumptions and simplifications made.	3,4
Critical appraisal of individual sources of evidence§	12	If done, provide a rationale for conducting a critical appraisal of included sources of evidence; describe the methods used and how this information was used in any data synthesis (if appropriate).	3,4
Synthesis of results	13	Describe the methods of handling and summarizing the data that were charted.	3,4
**RESULTS**			
Selection of sources of evidence	14	Give numbers of sources of evidence screened, assessed for eligibility, and included in the review, with reasons for exclusions at each stage, ideally using a flow diagram.	5
Characteristics of sources of evidence	15	For each source of evidence, present characteristics for which data were charted and provide the citations.	5
Critical appraisal within sources of evidence	16	If done, present data on critical appraisal of included sources of evidence (see item 12).	5

SECTION	ITEM	PRISMA-ScR CHECKLIST ITEM	REPORTED ON PAGE #
Results of individual sources of evidence	17	For each included source of evidence, present the relevant data that were charted that relate to the review questions and objectives.	6-13; Tables 1 and 2
Synthesis of results	18	Summarize and/or present the charting results as they relate to the review questions and objectives.	6-13
**DISCUSSION**			
Summary of evidence	19	Summarize the main results (including an overview of concepts, themes, and types of evidence available), link to the review questions and objectives, and consider the relevance to key groups.	8-13
Limitations	20	Discuss the limitations of the scoping review process.	23
Conclusions	21	Provide a general interpretation of the results with respect to the review questions and objectives, as well as potential implications and/or next steps.	23,24
**FUNDING**			
Funding	22	Describe sources of funding for the included sources of evidence, as well as sources of funding for the scoping review. Describe the role of the funders of the scoping review.	25

JBI = Joanna Briggs Institute; PRISMA-ScR = Preferred Reporting Items for Systematic reviews and Meta-Analyses extension for Scoping Reviews.

* Where *sources of evidence* (see second footnote) are compiled from, such as bibliographic databases, social media platforms, and Web sites.

† A more inclusive/heterogeneous term used to account for the different types of evidence or data sources (e.g., quantitative and/or qualitative research, expert opinion, and policy documents) that may be eligible in a scoping review as opposed to only studies. This is not to be confused with *information sources* (see first footnote).

‡ The frameworks by Arksey and O'Malley (6) and Levac and colleagues (7) and the JBI guidance (4, 5) refer to the process of data extraction in a scoping review as data charting.

§ The process of systematically examining research evidence to assess its validity, results, and relevance before using it to inform a decision. This term is used for items 12 and 19 instead of "risk of bias" (which is more applicable to systematic reviews of interventions) to include and acknowledge the various sources of evidence that may be used in a scoping review (e.g., quantitative and/or qualitative research, expert opinion, and policy document).

From: Tricco AC, Lillie E, Zarin W, O'Brien KK, Colquhoun H, Levac D, et al. PRISMA Extension for Scoping Reviews (PRISMAScR): Checklist and Explanation. Ann Intern Med. 2018;169:467–473. doi: 10.7326/M18-0850.
